# The effectiveness of interferon beta versus glatiramer acetate and natalizumab versus fingolimod in a Polish real-world population

**DOI:** 10.1371/journal.pone.0223863

**Published:** 2019-10-24

**Authors:** Katarzyna Kapica-Topczewska, Joanna Tarasiuk, Francois Collin, Waldemar Brola, Monika Chorąży, Agata Czarnowska, Mirosław Kwaśniewski, Halina Bartosik-Psujek, Monika Adamczyk-Sowa, Jan Kochanowicz, Alina Kułakowska

**Affiliations:** 1 Department of Neurology, Medical University of Bialystok, Bialystok, Poland; 2 Centre for Bioinformatics and Data Analysis, Medical University of Bialystok, Bialystok, Poland; 3 The Faculty of Medicine and Health Sciences, Institute of Physiotherapy, Jan Kochanowski University, Kielce, Poland; 4 Neurology Clinic with Brain Stroke Sub-Unit, Clinical Hospital No. 2 in Rzeszow, Medical Faculty, University of Rzeszow, Rzeszów, Poland; 5 Department of Neurology in Zabrze, Medical University of Silesia, Zabrze, Poland; University of Ioannina School of Medicine, GREECE

## Abstract

**Objective:**

The aim of the study was to assess the effectiveness of disease-modifying therapies (DMTs) in relapsing-remitting multiple sclerosis (RRMS) patients treated in MS centres in Poland.

**Methods:**

Demographic and clinical data of all Polish RRMS patients receiving DMTs were prospectively collected from 2014 to 2018 in electronic files using the Therapeutic Program Monitoring System (SMPT).

**Results:**

The study included 10,764 RRMS patients treated with DMTs in first-line and 1,042 in second-line programmes. IFNβ more effectively lengthened the times to the first relapse, disability progression, and brain MRI activity than GA. After 2 and 4 years of follow-up, more patients on IFNβ showed no evidence of disease activity (NEDA-3) in comparison to GA (66.3% and 44.3% vs 55.2% and 33.2%, respectively; p<0.001). NAT more effectively reduced brain MRI activity than FTY (p = 0.001). More patients under NAT had NEDA-3 after 2 and 4 years of follow-up compared to FTY (66.2% and 42.1% vs 52.1% and 29.5%, respectively; p = 0.03). In adjusted analysis, a higher baseline Expanded Disability Status Score (EDSS) was a predictor of relapse (p<0.001) and NEDA-3 failure (p = 0.003).

**Conclusion:**

IFNβ compared to GA and NAT compared to FTY more effectively reduced disease activity in a Polish population of RRMS patients.

## Introduction

Real-world studies provide new insight into MS therapy response, which is valuable to guide daily clinical practice [1]. Given many therapeutic options, the first therapy choice is challenging in RRMS and mainly depends on neurologist and patient preferences. In Poland patients with relapsing-remitting multiple sclerosis (RRMS) are treated with disease-modifying therapies (DMTs) at MS centres throughout country. The criteria for RRMS treatment are clearly described in drug programmes reimbursed by National Health Fund (Narodowy Fundusz Zdrowia, NFZ). The first- and second-line drug programmes were initiated in 2004 and 2013, respectively. The first-line programme includes: IFNβ since 2004, GA since 2005, pegylated interferon beta and dimethyl fumarate since 2016, and teriflunomide since 2017. Patients are allowed to change drugs within the first-line programme in the case of adverse effects or partial treatment failure (evidence of disease activity, but not fulfilling criteria for escalation to the second-line programme). Patients who do not respond to the first-line programme and meet the criteria for escalation are enrolled in the second-line programme with two available drugs since 2013: natalizumab (NAT) and fingolimod (FTY). Demographic and clinical data of all Polish RRMS patients receiving DMTs are prospectively collected in electronic files using the Therapeutic Program Monitoring System (*System Monitorowania Programów Terapeutycznych*, SMPT).

In Poland access to DMTs is strictly regulated by NFZ. In recent years, criteria for the first-line drug programme have been modified to increase access to this treatment, however modified criteria did not impact the results of the present study. But, due to restrictive criteria for escalation to the second-line, small number of patients have been treated with NAT and FTY. Because access to drug programs in Poland is stringent compared to other European countries, differences in effectiveness of DMTs are expected in comparison to studies conducted in other countries.

The objective of this study was to compare the effectiveness of DMTs under real-life conditions in a population of RRMS patients using both clinical and magnetic resonance imaging (MRI) outcomes. In a large-scale study, we compared the effectiveness of IFNβ versus GA in the first-line treatment and FTY versus NAT in the second-line treatment.

## Methods

This observational multi-centre study with prospective data collection was performed in a cohort of RRMS patients, diagnosed according to the 2010 McDonald criteria [2]. The study included all patients treated at MS centres throughout Poland who received DMTs reimbursed by NFZ. Poland has 128 and 59 MS centres for first- and second-line programmes, respectively. Only DMTs used in programme for more than 4 years were evaluated: IFNβ, GA for the first-line, FTY and NAT for the second-line. In first-line programme, patients must have experienced at least one relapse or show one new gadolinium (GD+) lesion on MRI in the preceding 12 months before the qualification and an Expanded Disability Status Scale (EDSS) score <5.0 point is needed. The IFNβ drug group included subcutaneous and intramuscular IFNβ 1a, and subcutaneous IFNβ 1b. Patients’ access to the second-line programme is allowed in absence of response to a complete 1-year cycle of DMT (minimum, first-line treatment), defined as the fulfilment of both of these two conditions:

1. two or more moderate relapses requiring administration of steroids (an increase of 1–2 points in EDSS score) or one severe relapse after 6 months of treatment (an increase in EDSS score higher than 2 points)
  and
2. minimum two GD+ lesions or three new T2-weighted lesions on MRI performed every 12 months of therapy.

In Poland, until 2018, only patients with negative John Cunningham virus (JCV) tests were treated with NAT due to drug national programme guidance. The JCVAb status was determined by STRATIFY JCV^™^ (performed at Unilabs, Copenhagen, Denmark) in all NAT-treated patients every 6 months.

Data were prospectively collected from 2014 to 2018 in the SMTP, developed and distributed by the NFZ to record electronic documentation of DMTs implementation and monitoring. The use of SMPT by neurologist to record medical care pathways of MS patients treated with DMTs is compulsory. Every patient registered in any MS centre across the country starting DMT was enrolled.

Patients eligibility and monitoring in both programmes relied on Expanded Disability Status Scale (EDSS), relapses and MRI activity. A relapse was defined as new or recurrent neurologic symptom not associated with fever or infection that lasted for at least 24 hours. Neurological examination including EDSS scoring was performed at baseline and then every 12 months. EDSS score worsening was confirmed after 12 months by treating neurologist with experience in MS care. Brain MRI was performed before treatment initiation, then every 12 months in the local MS centre and reviewed by the radiologist and treating neurologist with experience in MS care. Annual evaluations of effectiveness, based on results of neurological examination and MRI, determine whether a patient may continue on treatment. DMT should be terminated in cases of secondary progressive MS development. The study was approved by the Regional Medical Ethics Committee (the Medical University of Bialystok), and written consent to use the data for scientific research was obtained from the President of the National Health Fund. We used the anonymized registry data. Every patient undertaking treatment reimbursed by NFZ consented to the collection of data in SMPT. We have received from the NFZ anonymous data that prevents identification of the patient. The information collected did not cause harm to patients.

### Outcomes were

1. Time to first relapse, determined by an annual observation.
2. Percentage of patients without relapses and disability progression.
3. Time to disability worsening, defined as an EDSS score increase ≥1 point with baseline EDSS (the highest baseline EDSS was 5.0).
4. Time to brain MRI activity, defined as ≥1 new T2 lesion and/or ≥1 GD+ lesion with respect to previous brain MRI.
5. Duration of No Evidence of Disease Activity (NEDA-3: no relapses, no brain MRI activity, and no disability worsening), defined as the duration to first evidence of disease activity (either relapse or brain MRI activity) or disability progression.

Survival analysis or right-censored data was performed to investigate the expected duration from start of DMT to reported time of either relapse or disease progression or MRI activity (T2 or GD+ lesions) or NEDA-3 status failure. The study focused mostly on the four first years of treatment. However, as data were collected from 2014 to 2018, the patient observation time was uneven, from several months to several years. Therefore, if the events listed above had not been observed for a patient, which might be the case when the patient resigned from the program or was free from disease activity at the last visit, then the patient was not taken into account (“censored”) after the last valid observation. Survival estimations were therefore based on all available observations and every patient was monitored until event or censoring time. We compared survival time endpoints using multivariate proportional hazard Cox regression models. Effects of treatment were adjusted for age, sex, and EDSS at the start of treatment. Results are presented as hazard ratios (HR), p-values, and 95% confidence intervals (CIs) for HRs. Kaplan–Meier curves were generated to graphically demonstrate the survival rates of patients on different treatments. Kaplan-Meier curves were calculated with the software R (version 3.5.3, R Core Team, 2019). Analyses were performed in SPSS IBM v. 20.0 statistical software (IBM Corp., Armonk, NY, USA). Statistical hypotheses were verified at a significance level of 0.05.

## Results

### Patients’ characteristics

The initial extract from the SMPT electronic health record accounted for longitudinal monitoring of RRMS-care within the public health care system, gathering 49185 records. The records corresponded to 15368 prescriptions that were then individually described by prescription information (e.g. treatment line, drug prescribed, starting date), patient information (e.g. patient ID, age at DMT start), disease baseline data (e.g. EDSS and MRI information) and monitoring synthesis (e.g. duration of observation, first relapse date). Precautions were finally taken to remove aberrant or incomplete data, ensuring at least yearly evaluation of every patient × prescription, achieving a collection of 11926 prescriptions. These prescriptions corresponded to 10,764 and 1,042 patients with RRMS treated with DMTs in first- and second-line programmes, respectively. At baseline, the first-line programme included 7,603 females and 3,161 males, while the corresponding numbers for second-line were 685 and 357 males. At baseline, the female-to-male ratios were 2.40 and 1.92 in first and second-line, respectively. First-line programme patients were treated with IFNβ (77.6%) or GA (22.4%), and second-line patients received FTY (65.6%) or NAT (34.4%). As extracted from the in-production SMPT, data availability decreases with monitoring time. The analysis started with 11926 prescription description at baseline, and progressively decreased (7,870 monitored cases > 12 months; 2,874 monitored cases > 48 months, Table 1).

**Table 1:** Estimation corresponding to the Kaplan-Meier curves: outcomes in rows (relapse, EDSS increase, T2 and Gd+ lesions, NEDA-3) and treatment in columns. For each outcome and treatment, the initial number of patient is given in the header following DMT name; then for each time interval (12, 24, 36 and 48 months since the start of DMT) were reported: the number of patient still at risk (n.risk), number of patient for which the outcome was measured during the given interval (n.event) and the corresponding estimation of survival probability (surv., probability that the outcome has not happened) along with its standard error (std.err).

		INF (init n.risk: 8464)				GA (2422)				FTY (682)				NAT (358)			
Outcome	time	n.risk	n.event	surv	std.err	n.risk	n.event	surv	std.err	n.risk	n.event	surv	std.err	n.risk	n.event	surv	std.err
Relapse	12	5603	613	0.91	0.0035	1172	247	0.855	0.0086	403	62	0.88	0.014	181	29	0.884	0.02
	24	4251	334	0.85	0.0045	777	90	0.78	0.011	251	46	0.765	0.02	107	13	0.808	0.027
	36	3124	241	0.796	0.0054	519	70	0.697	0.013	119	18	0.69	0.025	42	9	0.72	0.037
	48	2215	190	0.74	0.0064	347	42	0.632	0.016	32	6	0.637	0.031	8	1	0.695	0.043
dEDSS	12	5909	202	0.969	0.0021	1275	64	0.959	0.005	442	13	0.974	0.0072	196	7	0.971	0.011
	24	4502	262	0.922	0.0035	844	71	0.897	0.0086	300	20	0.922	0.013	123	5	0.939	0.018
	36	3304	243	0.865	0.0048	585	46	0.84	0.011	158	12	0.879	0.018	56	8	0.865	0.03
	48	2319	212	0.8	0.0062	379	45	0.764	0.015	47	7	0.831	0.024	9	3	0.796	0.048
T2	12	5492	750	0.885	0.0039	1136	294	0.813	0.0099	399	64	0.87	0.015	191	16	0.932	0.016
	24	4058	574	0.785	0.0053	721	152	0.688	0.013	251	53	0.741	0.021	113	12	0.862	0.025
	36	2966	377	0.703	0.0062	503	70	0.611	0.014	128	23	0.652	0.026	52	4	0.826	0.03
	48	2089	281	0.627	0.007	321	62	0.521	0.016	34	12	0.563	0.033	7	2	0.74	0.063
Gd	12	5831	318	0.951	0.0027	1224	160	0.898	0.0077	422	31	0.937	0.011	199	2	0.992	0.006
	24	4438	282	0.9	0.0039	803	85	0.823	0.011	282	26	0.87	0.016	121	4	0.967	0.014
	36	3279	190	0.856	0.0049	547	61	0.75	0.013	147	8	0.837	0.02	57	0	0.967	0.014
	48	2332	150	0.809	0.0059	367	24	0.711	0.015	42	8	0.773	0.029	11	1	0.928	0.04
NEDA-3	12	5049	1325	0.802	0.0049	1017	485	0.707	0.011	350	129	0.746	0.019	170	46	0.814	0.025
	24	3598	817	0.663	0.006	631	200	0.552	0.013	187	95	0.521	0.024	95	27	0.662	0.033
	36	2546	563	0.55	0.0066	418	120	0.436	0.014	81	35	0.394	0.026	37	14	0.541	0.04
	48	1729	450	0.443	0.007	257	88	0.332	0.014	22	15	0.295	0.03	4	4	0.421	0.063

Patients' characteristics were shown in Table 2.

**Table 2:** Patients' characteristics.

	INF	GA	FTY	NAT
number	8464.00	2422.00	682.00	358.00
sex ratio F:M	2.37	2.53	1.77	2.25
age at DMT start	35.00	37.00	35.00	33.00
median EDSS (baseline)	1.50	2.00	3.00	3.50
median from the first symptoms to MS diagnosis (months)	7.20	8.20	5.00	5.50

### DMT effectiveness

#### Relapses

Amongst patients who received IFNβ, 947 of them (613 after 12 months and 334 after 24 months, Table 1) had a relapse in the 24 months following the date of starting treatment with DMT; given censored patients during that interval, relapse-free percentage at 24 months was estimated as high as 85.0%. By comparison, the percentage of relapse-free patients at 24 months for GA was 78.0%, FTY 76.5% and NAT 80.8%. The relapse-free percentage decrease comparably across treatment until 48 months when it reached: 74.0% (IFNβ), 63.2% (GA), 63.7% (FTY) and 69.5% (NAT).

**Fig 1:**
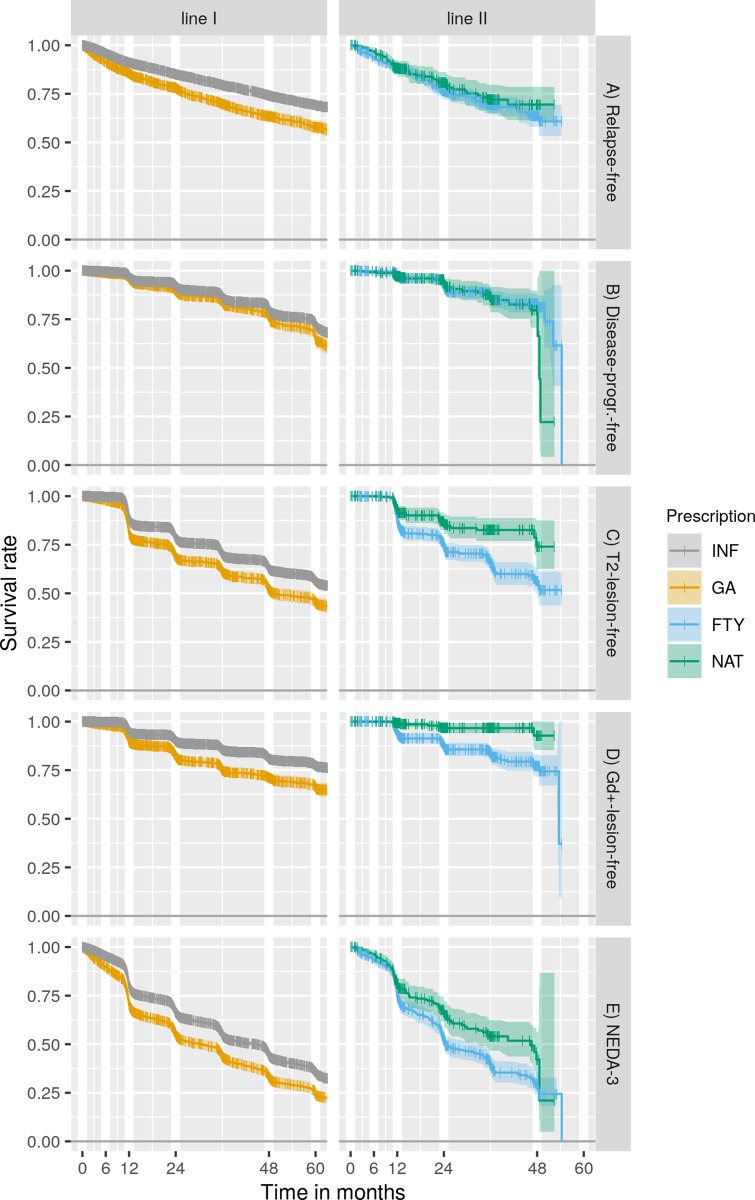
KM survival curves. A: Relapse-free survival rate B: Disease-progression-free survival rate progression C: T2-lesion-free survival rate D: Gd+-lesion-free survival rate E: NEDA-3 survival rate. The large confidence interval beyond 48 months observed in B, D and E in second-line treatment was due to algorithm artefact linked to low observation availability. Abbreviations: KM, Kaplan–Meier; INF, interferon beta; GA, glatiramer acetate; FTY, fingolimod; NAT, natalizumab; Gd+, gadolinium enhancement; NEDA-3, no evidence of disease activity.

In adjusted analyses, a higher baseline EDSS score was predictor of relapses for both programmes (p<0.0001). In first-line programme, the risk of the relapse decreased with age (HR: 0.989, 95% CI 0.984–0.994, p<0.001). IFNβ more effectively lengthened the time to first relapse compared to GA (HR: 0.631, 95% CI 0.569–0.700, p<0.000). No significant difference in time to first relapse was detected between NAT and FTY (Fig 1A).

#### Disability progression

Among first-line programme patients, 80.0% treated with IFNβ and 76.4% with GA did not experience disability progression at 48 months, while accounting for censored patient. In the second-line programme, 83.1% of FTY-treated patients and 79.6% of NAT-treated patients had no disability progression at 48 months.

In adjusted analysis, older age was a predictor of disability worsening in the first-line programme (HR: 1.034, 95% CI 1.026–1.043; p<0.001). IFNβ more effectively lengthened the time to disability progression compared to GA (HR: 0.610, 95% CI 0.515–0.723, p<0.001). Time to disability worsening was not different between NAT and FTY (Fig 1B).

#### Brain MRI activity

The probabilities of new T2 lesions within 48 months of therapy under IFNβ and GA treatments were 37.3% and 47.9%, respectively. By comparison, under FTY and NAT treatment, the probabilities of new T2 lesions within 48 months were 43.7% and 26.0%, respectively. Likewise, percentage of patient presenting new GD+ lesions during 48 months of treatment were 19.1%, 28.9%, 22.7% and 7.2% of patients treated with IFNβ, GA, FTY and NAT, respectively.

In adjusted analysis, the risk of new T2 and GD+ lesions decreased with age for patients in both programmes. In the second-line programme, higher baseline EDSS was associated with a lower risk of new GD+ lesion (HR: 0.778, 95% CI 0.657–0.922, p = 0.04). Time without brain MRI activity was longer in IFNβ compared to GA (p<0.001) and in NAT compared to FTY (p<0.001)(Fig 1C and 1D).

#### NEDA-3

NEDA-3 rates in the first-line programme after 24 and 48 months were as follows: IFNβ 66.3% and 44.3%, GA 55.2% and 33.2%. In the second-line programme, the rates were NAT 66.2% and 42.1% and FTY 52.1% and 29.5%. Patients treated with IFNβ were more likely to have NEDA-3 compared to GA (HR: 0.696, 95% CI 0.647–0.750; p<0.001). Patients treated with NAT were more likely to have NEDA-3 compared to FTY (HR: 0.690, 95% CI 0.0541–0.880; p = 0.003). In adjusted analysis, predictors of NEDA-3 failure were a lower age in both programmes and a higher baseline EDSS score in the first-line programme (HR: 1.063, 95% CI 1.032–1.094; p = 0.003) (Fig 1E).

**Fig 2:**
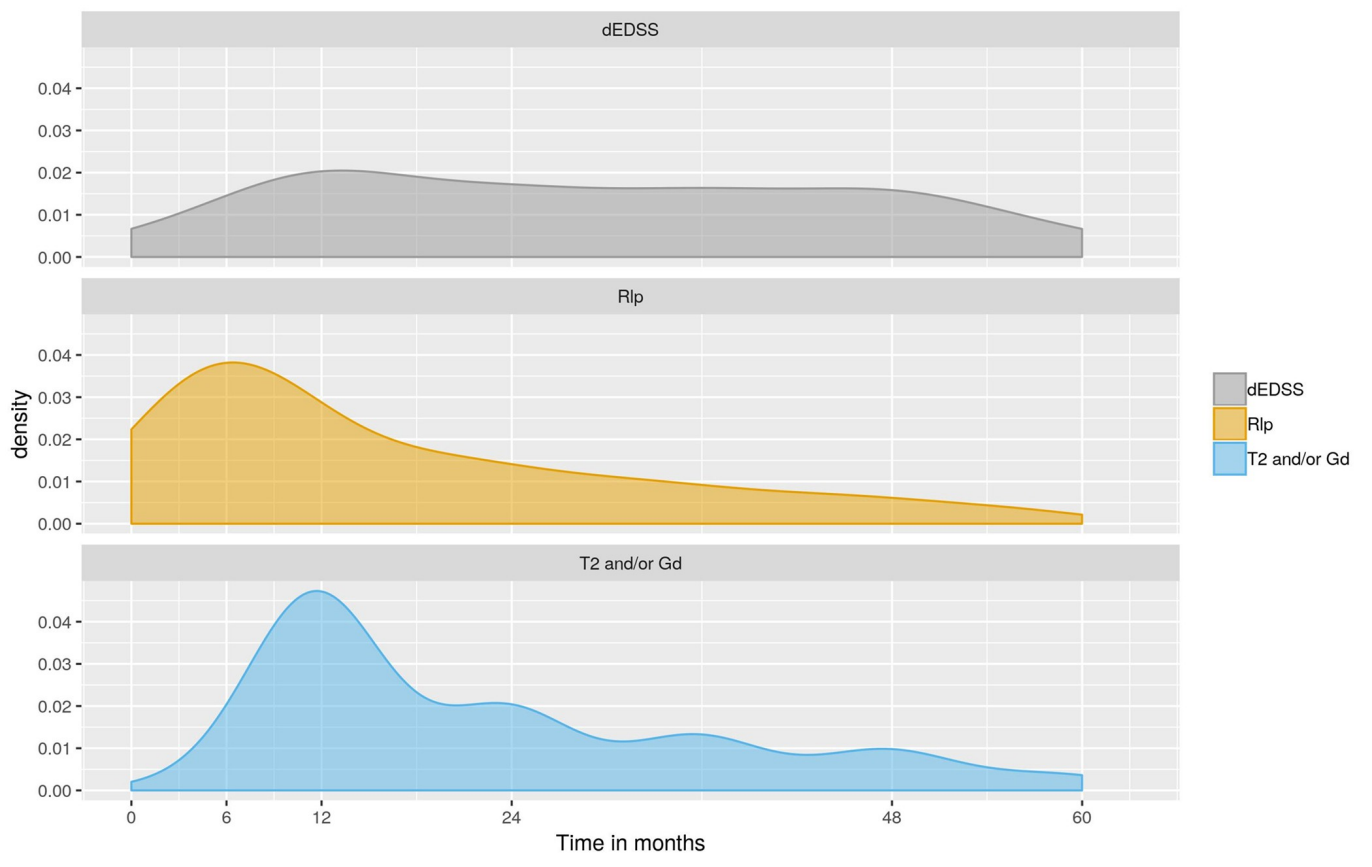
Density of causes for NEDA-3 failure. Abbreviations: NEDA-3, no evidence of disease activity.

Among the 4690 patients exhibiting disease activity (NEDA-3 status failure) within 60 months of treatment with DMTs, 4609 were uniquely associated with either relapse (1702) or EDSS increase (612) or MRI activity (new T2 or GD+ lesion, 2295). The density of occurrence of these monitored events was estimated to investigate how the cause for disease activity varies with the time (Fig 2). Results indicated that the highest frequency of relapse as the first evidence of disease activity occurred 6 months after starting treatment with DMT and then progressively decreased. Likewise, the first evidence of disease activity associated with MRI activity exhibited a peak at 12 months and then progressively decreased. Noting that relapse were retrospectively recorded and associated with the actual date of relapses while MRI activity data was mostly acquired on a yearly basis (±6months), this difference in peak would rather be attributed to methodological constraint while and no differences in peak time could be deduced. This was also evidenced by the smooth relapse distribution compared to the yearly wavelets visible on MRI data distribution. Therefore, relapse and MRI activity followed a very comparable distribution over time since the start of treatment. In comparison, the distribution of disease progression revealed a relative independence with the duration of treatment.

## Discussion

Post-marketing real-world studies which are based on actual patient records provide complementary data to clinical trials and allow long-term insight into drug effectiveness and safety profiles [1]. Currently, 13 approved DMTs with different mechanisms of action and effects on the immune system are available to treat MS worldwide. Limited data are currently available that directly compare multiple drugs and DMT selection in real clinical practice remains empirical. Although based on incomplete benefit–risk assessments and potentially unknown long-term safety risks, neurologists seek to individualise and optimise the therapies. The strength of this study was that the effectiveness of several DMTs with multiple outcomes when administered to a large cohort of patients in clinical practice over a relatively long follow-up period was analysed. Our results confirmed good effectiveness of DMTs as most of the treated patients remained free from relapse, disability progression, and MRI disease activity during 48 months. Considering all outcomes, the most important predictor of disease activity was baseline EDSS score, which was related with relapses and NEDA-3 status failure. Other authors have also reported a relationship between EDSS and disease activity [3–5]. In our study, the risk of disability progression increased with age, but the risks of relapse and NEDA-3 status failure went down. The effects of ageing on the immune system manifest at multiple levels that include reduced production of B and T cells in bone marrow and thymus and diminished function of mature lymphocytes in secondary lymphoid tissues [6]. The inflammatory process decreases with age, but neurodegenerative processes are more likely to occur. Additionally, comorbidities and concomitant medications are increasing with age, therefore in older MS patients; therapies should be chosen considering favourable safety profiles [7]. INFβ and GA are widely used by patients with RRMS. Many studies confirmed that long lasting IFNβ and GA treatment protects against disability worsening and support a beneficial effect on long-term disability [3,8–14]. However, it is still unclear whether they have different effectiveness in real-world studies. The efficacy of INFβ and GA was investigated in five randomised trials which included 2904 RRMS patients [8]. It was evidenced that both therapies do not to differ in terms of clinical efficacy and safety, although IFNβ was found to limit the increase of MRI lesion burden more effectively than GA. But, all studies were at high risk for attrition bias [8]. Another meta-analysis [9] which identified 24 randomised trials, reported in 42 publications, confirmed that IFNβ and GA reduce annual relapse rate and generally delay progression as defined in these trials, but there was no clear 'winner' across outcomes. Nevertheless, most studies were at high risk of bias in at least one domain [9]. Similar clinical outcomes were confirmed in long-term follow-up study of MS patients initially treated with IFNβ and GA [14]. In our real-world study, in first-line programme IFNβ more effectively lengthened the times to relapse, disability progression, brain MRI activity, and prevented NEDA-3 status failure in comparison to GA.

The second-line programme is restricted to NAT and FTY and is only available to patients who suffered from relapses and simultaneously demonstrated new lesions on MRI. Restricted number of patients with disease activity treated in the first-line programme meets these criteria; only patients with severe relapses and radiological disease activity with evidence of GD+ >1 or T2 lesions >2 on brain MRI. Therefore, some patients with NEDA-3 status failure can only change treatment within the first-line programme. Several observational studies have compared the effectiveness of NAT and FTY, but their results are inconsistent [3,15–23]. We found that NAT is more effective than FTY at reducing brain MRI activity and increasing the number of patients with NEDA-3 status. No difference was found in relapse rate or disability progression. In Poland, due to restrictive criteria of the second-line programme, population of MS patients treated with NAT and FTY had more active course of disease than MS patients living in Western Europe, the effects of these therapies on disability progression were similar to European studies [3,17–18, 22]. Although a study evoked that NAT was more effective in improving disability compared with FTY, no differences in the proportion of patients free from EDSS progression was observed [23]. We also found that switching to NAT and FTY after first-line injectable therapies led to a reduction of clinical and radiological disease activity [3, 11, 22–26]. Cohort study using MSBase, identified patients with RRMS experiencing relapses or disability progression within the 6 months immediately preceding switch to either NAT or FTY. No difference in the rate of sustained disability progression events was observed between the groups, but the change in overall disability burden (quantified as area under the disability-time curve) was lower in NAT group than FTY. This study suggests that in active multiple sclerosis during treatment with injectable DMTs, switching to NAT is more effective than switching to FTY in reducing relapse rate and short-term disability burden [22].

Real-world studies have inherent limitations such as confounding factors. In a certain extent, the main known confounding factors of our study were accounted for in the CoxPH models.

The strength of this study is that it was conducted on a large homogeneous population including all patients treated in Poland. Indeed, this country having a population of over 38.5 million people (the sixth most populous member of the European Union), is also ethnically homogeneous, which is expected to reduce importance of inter-ethnics biases [27].

Due to the limitations of drug programmes, access to DMTs in Poland is one of the lowest in Europe. It is not only lower than those found in highly developed countries (Germany, the Scandinavian countries), but also in comparison to the countries of Central Europe, for example in the Czech Republic [27]. In Poland, access to DMT is strictly regulated by the NFZ, which significantly limits the second-line treatment. Only 8.8% of all Polish patients treated with DMTs were using NAT and FTY. Our findings showed high effectiveness of the second-line DMTs, which stress the health benefits of facilitated access to these therapies; less restrictive criteria for switching to the second-line programme should be established.

In our study, patients without disease activity (i.e. fulfilling NEDA criteria) constitute a small proportion of all treated group, especially in the long-term follow up. Unquestionably, ‘no evidence of disease activity’ is the main goal for MS treatment, but demonstrating the utility of NEDA as a biomarker requires long-term observation [28–29]. Long-term follow-up from the randomized trial of IFNβ-1b permitted the assessment of different definitions of NEDA for predicting long-term outcome in MS. In this study clinical NEDA predicted long-term disability outcome. By contrast, definitions of NEDA that included on-therapy changes in MRI variables did not increase the predictive validity [30]. Our results showed that relapse and MRI activity as the first evidence of disease activity occurred 6–12 months after starting treatment with DMT and then progressively decreased. In comparison, disease progression was relatively independent from treatment duration. This observation was confirmed by Rotstein et al. who observed that the proportion of disease-progression-free patient decreased linearly with time while both proportion of relapse- and MRI-activity-free patient followed an exponential decreasing function [31]. Mathematically, this indicated that the disease progression prevalence as a cause of NEDA-status failure increased with the time. So, during the long-term follow up a greater proportion of MS patients lose NEDA-status based on clinical rather than MRI criteria.

## Conclusion

1. A high proportion of RRMS patients treated with DMTs achieves relapse free status, reduction in disability progression and MRI disease activity.
2. The higher EDSS score at baseline was a predictor of relapses and NEDA-3 status failure.
3. The age is a risk factor of disability progression.
4. IFNβ more effectively lengthened the times to first relapse, disability progression, brain MRI activity and NEDA-3 status failure as compared to GA.
5. NAT more effectively than FTY reduced brain MRI activity and NEDA-3 status failure.
6. NAT and FTY reduced clinical and radiological disease activity after the switch from first-line injectable therapies.
7. In Poland less restrictive criteria for switching to the second-line treatment should be established to increase patients access to more effective therapies.

## Supporting information

S1 Table Survival data–anonymised dataset. (CSV)
